# Curvature of Double-Membrane Organelles Generated by Changes in Membrane Size and Composition

**DOI:** 10.1371/journal.pone.0032753

**Published:** 2012-03-12

**Authors:** Roland L. Knorr, Rumiana Dimova, Reinhard Lipowsky

**Affiliations:** Max Planck Institute of Colloids and Interfaces, Science Park Golm, Potsdam, Germany; Institut Curie, France

## Abstract

Transient double-membrane organelles are key players in cellular processes such as autophagy, reproduction, and viral infection. These organelles are formed by the bending and closure of flat, double-membrane sheets. Proteins are believed to be important in these morphological transitions but the underlying mechanism of curvature generation is poorly understood. Here, we describe a novel mechanism for this curvature generation which depends primarily on three membrane properties: the lateral size of the double-membrane sheets, the molecular composition of their highly curved rims, and a possible asymmetry between the two flat faces of the sheets. This mechanism is evolutionary advantageous since it does not require active processes and is readily available even when resources within the cell are restricted as during starvation, which can induce autophagy and sporulation. We identify pathways for protein-assisted regulation of curvature generation, organelle size, direction of bending, and morphology. Our theory also provides a mechanism for the stabilization of large double-membrane sheet-like structures found in the endoplasmic reticulum and in the Golgi cisternae.

## Introduction

**Figure 1:**
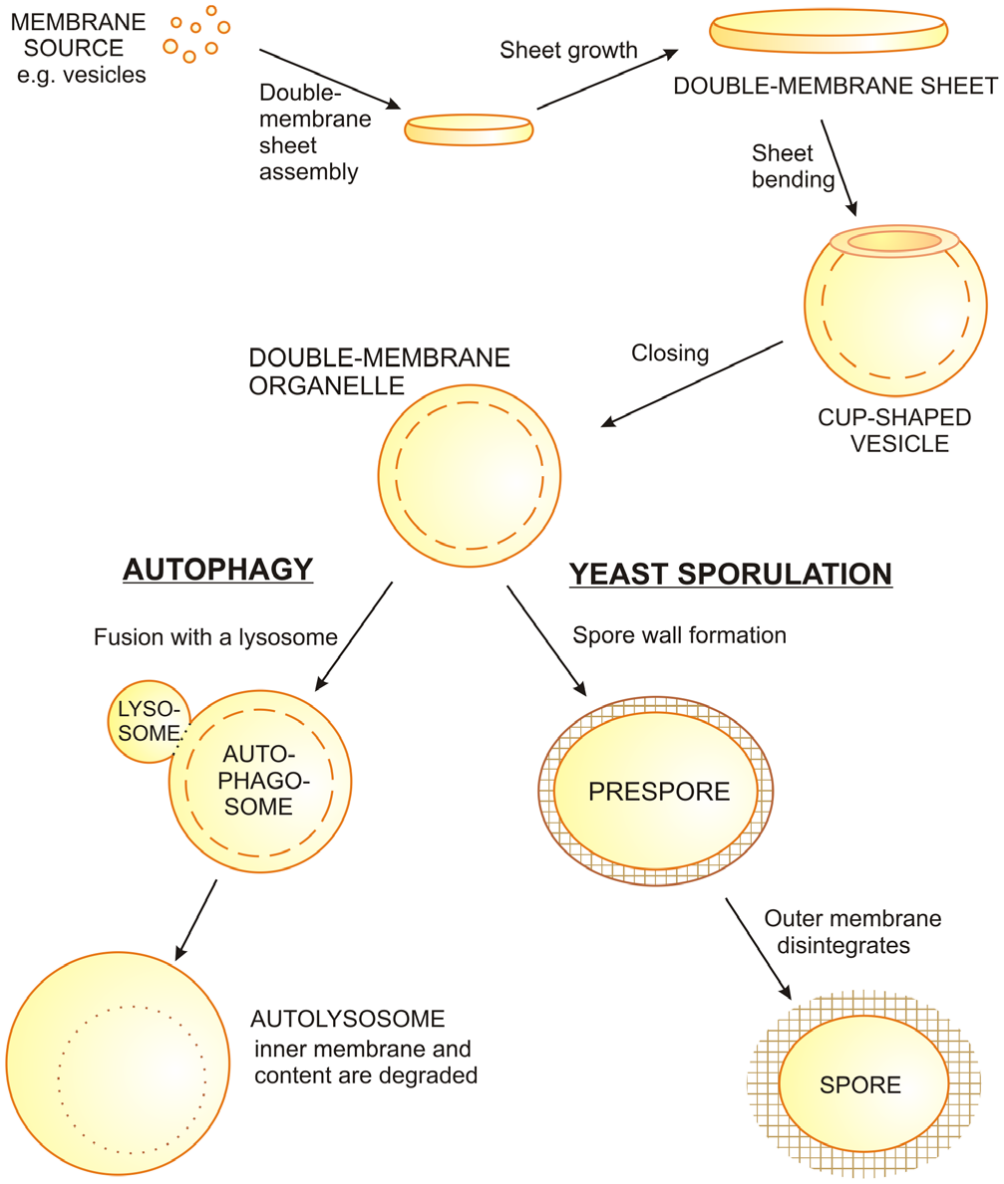
Growth and fate of cup-shaped vesicles in cells. Double-membrane sheets can be built by fusing small vesicles. The sheets grow to a critical size, bend and eventually close to form a double-membrane organelle, for intermediate states see Fig. 2. In the autophagosomal pathway, autophagosomes fuse with lysosomes. In the resulting autolysosome, the cytosolic content of the autophagosome becomes degraded together with the inner vesicle membrane. In the yeast sporulation pathway, a spore wall is synthesized between both membranes to build the prespore. The completed spore has only one membrane, because during spore wall assembly the outer membrane disintegrates.

Eukaryotic cells contain a variety of organelles, some of which consist of an assembly of extended double-membrane sheets such as in the endoplasmic reticulum, while others are enclosed by two bilayer membranes. Double-membrane organelles (DMOs) can be either permanent or transient. One example for permanent DMOs is provided by mitochondria. Transient organelles with a double membrane are formed during specific stages of cell life: examples are autophagosomes that form during macroautophagy (autophagy hereafter) [1], and forespore membranes that assemble during sporulation of yeasts [2], see Fig. 1. Double-membrane vesicles are also formed when cells are infected by plus-stranded RNA viruses [3]. These three examples will be briefly reviewed in the following.

- Autophagy is a membrane-mediated intracellular degradation process, where parts of the cytoplasm are sequestered by bending flat, double-membrane sheets (phogophores) into DMOs, the autophagosomes. At a later stage, the autophagosomes fuse with lysosomes, where the autophagosomal content eventually degrades, see Fig. 1. Autophagy is essential for cell survival under basal conditions or stress, for the control of embryonic and postnatal development, for immunity, tumorigenesis, aging and neurodegenerative disorders [1], [4], [5].
- During sporulation or gametogenesis in yeast, the meiotic division splits the diploid nucleus into four haploid nuclei, which become enwrapped by newly formed forespore (or prespore) membranes [2]. A short meiotic spindle assembles between the inner sides of both spindle pole bodies, while at their outer surfaces the forespore membranes grow. These double membranes curve and close into DMOs, the prespores, see Fig. 1.
- During infection, plus-stranded RNA and some DNA viruses induce certain remodeling of the cytoplasmic membrane ensuring the replication of the virus genome. In the course of this process, paired membranes derived from the endoplasmic reticulum (ER) bend and close into double-membrane vesicles [3], [6], similarly to autophagy and spore formation.

Despite their very different cellular functions, the generation of autophagosomes and prespore membranes share many similarities. Both organelles are formed *de novo* if required, play a critical role in cell survival [1], [7] and exhibit similar steps during their morphological transitions. At first, cells start growing flat, disk-like double-membrane sheets. These sheets bend into cup-like intermediates and close into spherical DMOs. In addition to cytoplasm, these new organelles may encapsulate the nucleus (sporulation) or ingest specific cargoes such as damaged organelles or invasive microbes (autophagy), see Fig. 1.

How double-membrane sheets are forced to bend into spherical organelles is not understood. Likewise, the cellular regulation mechanisms of this curvature generating process are not known. Detailed understanding of both of these aspects is fundamental for unraveling the processes of autophagy [4], [8], [9], sporulation [2], and membrane remodeling induced by viruses [3], [6].

Here, we show that the formation of transient double-membrane organelles may be driven predominantly by the elastic properties of the membrane. We start from the bending energy of the membrane, which depends on its curvature [10], [11], and determine this energy for double-membrane sheets and vesicles as well as for their cup-shape intermediates. This energy landscape depends strongly on the size of the sheet, i.e., on its total membrane area. Above a certain critical size, the double-membrane sheet becomes unstable and undergoes a transition to a double-membrane vesicle. This critical size depends primarily on two properties both of which can be dynamically regulated by proteins: the preferred or spontaneous curvature of the membrane at the sheet rim, and a possible asymmetry between the two faces of the sheet. Furthermore, the critical sheet size is found to exhibit a sharp maximum as a function of the preferred rim curvature and, thus, to be very sensitive to small changes in this curvature.

We propose that in cells, the same mechanism is responsible for the formation of transient DMOs. We also identify regulatory mechanisms for such a shape transition. For example, we consider the effect of membrane adhesion on specific autophagy and point out other mechanisms by which proteins and lipids can be used to adjust physiologically relevant parameters such as the direction of sheet bending and the final size of the organelles. Qualitative agreement of the theoretical predictions with available experimental data emphasizes the relevance of the proposed mechanisms for autophagy and sporulation. More generally, our theory shows that the stability of extended sheets requires an “up-down” symmetry between the two faces of the double-membrane sheets, i.e., the outer leaflets of the two apposing membranes must be similar in their structure and composition and likewise for the two inner leaflets. This condition is not only important for the generation of very large transient double-membrane organelles, but may also be crucial for stabilizing sheet-like DMOs, such as parts of the ER and the cisternae of the Golgi apparatus.

## Methods

### Bending energy minimization

All cellular membranes are in a fluid state. The shape of vesicles composed of such fluid membranes is primarily governed by bending elasticity. On the micrometer scale, the bending energy of a membrane with uniform composition depends only on a few elastic parameters. This mesoscopic description has been corroborated by a detailed and quantitative comparison between experimentally observed and theoretically calculated shapes [10], [11].

The bending energy of any material is governed by its bending rigidity (or elastic modulus) κ, which has the units of energy. Fluid bilayers are very flexible and their rigidity κ is on the order of 10–20 *k_B_T*, where *k_B_* is the Boltzmann constant and *T* the temperature.

The curvature of any surface can be described locally by two perpendicular arcs. The inverse radii of these arcs are the two principal curvatures, which characterize the local shape of a membrane. The arithmetic mean of both curvatures defines the mean curvature *M* of a bilayer. If the two leaflets of the bilayer membrane differ in their molecular composition, the membrane has a certain preferred (or spontaneous) curvature *m*. The bending energy *E* of a vesicle with area *A* has the form [10]:
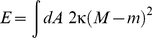
(1)where the integral is over the entire membrane surface. For symmetric bilayers, the preferred curvature can be taken to be zero but may change if proteins bind asymmetrically to the membrane or when the lipid composition of one of the bilayer leaflets changes.

**Figure 2:**
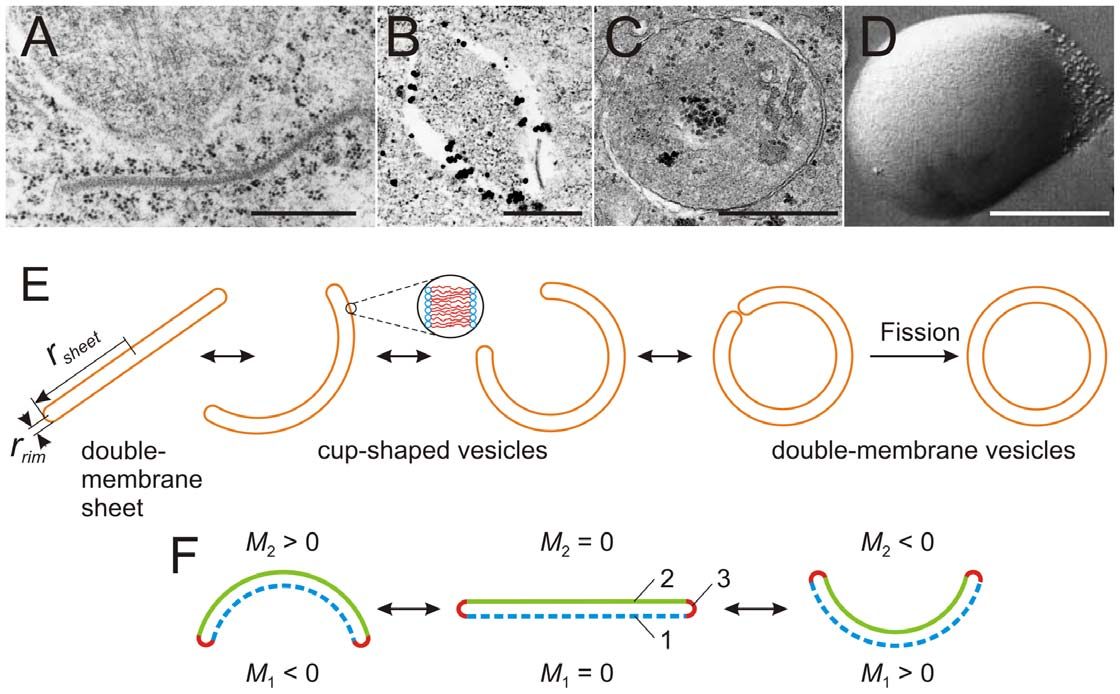
Cellular double-membrane organelles at different stages of their genesis. (A–D) electron microscopy micrographs, (E) schematic illustration of the sequence of shape changes, and (F) schematic cross section of the sheet with possible bending directions. (A) A growing double-membrane sheet during spore formation in *Schizosaccharomyces pombe*, [2]. (B) A cup-shaped phagophore with immunogold label (black spots) for the mammalian Atg8 homologue GATE16 [51]. (C) A closed autophagosome with the double membrane clearly visible [50]. (D) A freeze-fracture electron micrograph showing the smooth autophagosomal membrane. In the upper right corner a small, particle-rich endosome had fused with the autophagosome [47]; the smooth surface of the autophagosome away from the fusion area suggests the absence of a protein coat. All scale bars correspond to 0.5 µm. The electron microscopy images were adapted with permissions of the J. Cell Sci. and Elsevier. (E) Schematic illustrations of the shape transition from a double-membrane sheet to a double-membrane vesicle (cross sections shown). The solid line represents one bilayer. Geometrical parameters used in the main text are indicated in the first cartoon. The transition between the flat sheet and the vesicle can be reversible. The final step of generating the double-membrane vesicle requires irreversible fission. (F) Schematic cross sections of the sheet and cup-shape morphologies. Three different segments of the shapes are distinguished: lower segment (1, dashed blue), upper segment (2, solid green), and highly curved rim (3, solid red). The sheet (middle) is characterized by zero mean curvatures of the upper and lower segments, *M*_1_ = *M*_2_ = 0. When the sheet bends downwards (left), the mean curvature of the lower segment is negative, *M*_1_<0, and that of the upper segment is positive, *M*_2_>0. The situation is reversed when the sheet bends upwards (right).

Figure 2A–D shows electron micrographs illustrating different stages in the genesis of transient double-membrane structures found during autophagy and sporulation in cells [12], [13]. The characteristic morphological transitions are schematically shown in Fig. 2E. We consider the two limiting shapes of a circular, double-membrane sheet and a double-membrane sphere, and calculate the bending energies of these shapes and their cup-shaped intermediates. In order to take into account that the preferred curvature of the membrane can be inhomogeneous resulting, for example, from local adsorption of molecules, we will distinguish three different zones of the starting shape of a double-membrane sheet: the upper and lower flat parts and the curved edge; see Fig. 2F. These different segments may have different preferred (or spontaneous) curvatures *m*_1_, *m*_2_ and *m*_3_, respectively. The bending energy of the double membrane is then given by
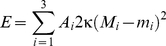
(2)where *A_i_* and *M_i_* are the area and mean curvature of segment *i*, respectively.

In general, the elastic energy density of the membrane also includes a term proportional to the Gaussian curvature of the membrane surface [14]. The corresponding elastic parameter is the so-called Gaussian curvature modulus κ_G_. During the closure of the double-membrane disk into a double-membrane spherical organelle, the energy contribution from the Gaussian curvature term is constant and equal to 4πκ_G_ as long as the rim forms a narrow neck that connects the two membranes. After the fission of this neck, the Gaussian curvature term contributes 8πκ_G_ to the elastic energy. Since κ_G_ is expected to be negative, the fission process will lower the double-membrane energy by 4πκ_G_. In the following, we will focus on the stability and the closure of double-membrane sheets, for which the Gaussian curvature term plays no role.

### Double-membrane sheets

A double-membrane sheet, which is initially flat, is characterized by two geometrical parameters: its lateral dimension is defined by the radius *r_sheet_* and its thickness or interbilayer distance by 2*r_rim_*, see Fig. 2E. The lateral dimensions of the double-membrane sheets in cells are typically much larger than their thickness, *r_sheet_*≫*r_rim_* (see e.g. Fig. 2). We first consider the simple case of a homogeneous symmetric membrane with vanishing preferred curvature, i.e., *m*_1_ = *m*_2_ = *m*_3_ = 0. The flat parts of the sheets have zero mean curvature (*M* = 0), and thus, do not contribute to the total bending energy of the sheet, *E_sheet_*. The only contribution to be considered is the rim energy of the sheet arising from the strongly curved membrane. The rim curvature depends on the sheet and rim radii and the mean curvature along the rim is *M* = (1/*r_rim_*+1/*r_sheet_*)/2. Thus, one obtains the sheet energy [15]

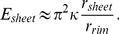
(3)


Note that this sheet energy can be rewritten as a product of the sheet's circumference, which is equal to 2π*r_sheet_*, times the effective rim tension πκ/2*r_rim_*.

Certain molecules such as proteins with BAR-domains or lipids like PI(3)P can bind specifically to the rim. Such an adsorption process will typically induce a preferred or spontaneous curvature *m*_3_>0 of the rim. Small values of *m*_3_ always act to reduce the sheet energy *E_sheet_*, see Equation 1 in Text S1. Furthermore, the effective rim tension depends on the rescaled curvature *m*_3_ *r_rim_* and vanishes for *m*_3_ *r_rim_* = 1/2, see Equation 5 in Text S1. This effect will be discussed further below.

### Double-membrane vesicles or spherical organelles

We now consider double-membrane vesicles or spherical organelles, for which we can distinguish two different states. The first state arises from the closure of the double-membrane sheet, after which the two membranes form two concentric, spherical shapes, which are still connected by a small membrane neck. This neck undergoes a fission process and breaks up, which leads to two separate membranes, each of which contains a small membrane pore. After the closure of these pores, the double-membrane vesicle attains its second state corresponding to two concentric, spherical membranes, which are no longer connected, see Fig. 2E. These two states have very similar bending energies as described by Eq. (1). Indeed, the two states differ only by the presence or absence of the small neck, which represents a saddle-like structure. The two principal curvatures of a saddle have opposite signs, and the mean curvature is approximately zero, *M*≈0. In addition, the neck occupies only a very small membrane area. Therefore, we can ignore the bending energy of the neck which implies that the bending energy *E_ves_* of a closed double-membrane vesicle or spherical organelle has the simple form
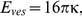
(4)as follows from Eq. (1).

For non-zero preferred (or spontaneous) curvature, the expression for the bending energy is given by Equation 2 in Text S1. Equation (4) implies that the bending energy of the double-membrane *organelle* does not depend on its size. In contrast, the bending energy of a double-membrane *sheet* increases with the sheet size *r_sheet_*, see Eq. (3). For sufficiently large sheets, the energy of a double-membrane organelle with the same area becomes smaller than that of a sheet, *E_ves_*<*E_sheet_*, and the sheet-like morphology will no longer represent the state of lowest bending energy.

## Results and Discussion

### Energy landscape of double-membrane shapes

**Figure 3:**
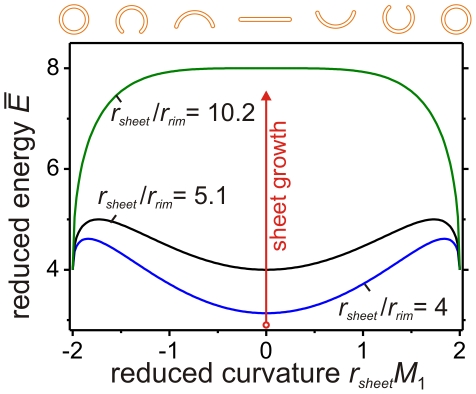
Reduced bending energy of double-membrane shapes, 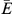
 **, as a function of the reduced curvature** ***r_sheet_M*** **_1_.** The results are calculated for vanishing preferred or spontaneous curvatures *m*_1_ = *m*_2_ = *m*_3_ = 0 and vanishing curvature asymmetry *m*_12_ = 0; see Equation 8 in Text S1 for the definition of 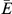. The reduced curvature *r_sheet_M*_1_ of the cup shapes can be positive or negative, which distinguishes between upward and downward bending of the sheet as schematically illustrated in the top row of the figure. For *r_sheet_*/*r_rim_*<5.1 the sheet represents the shape of minimal energy. At *r_sheet_*/*r_rim_* = 5.1 the flat sheet and the closed double-membrane vesicle are local minima with the same energy, but separated by a considerable energy barrier preventing the shape transition. Increasing the effective size of the vesicle decreases the barrier continuously. At the critical size, *r_sheet_*/*r_rim_* = 10.2, the energy barrier disappears and the sheet becomes unstable with respect to arbitrarily small perturbations, which transforms the sheet into a closed vesicle. Energy landscapes of asymmetric sheets with nonzero curvature asymmetry *m*_12_ are displayed in Fig. S3.

We first describe the evolution of the energy landscape for a double-membrane sheet with variable size *r_sheet_* and vanishing preferred curvature, see Fig. 3. The details of the corresponding calculations are described in Text S1. The bending and closure of the double-membrane sheet can proceed towards either sides of the sheet. The different morphologies adopted by the double membrane are illustrated in the first row of Fig. 3. The mean curvature *M*_1_ of the lower membrane segment is negative for a cup-like shape that bends downwards, vanishes for the sheet, and is positive for a cup-like shape that bends upwards, compare Fig. 2F.

For a small sheet with *r_sheet_*/*r_rim_*<16/π≈5.1, the flat double-membrane sheet has a lower bending energy than the double-membrane organelle. When the sheet size has attained the value *r_sheet_*/*r_rim_*≈5.1, the double-membrane organelle has the same bending energy as the double-membrane sheet but the two states are separated by an appreciable energy barrier, Δ*E* = 4πκ. Lipid membranes have bending rigidities in the range κ = (10–20)*k_B_T* [16], which implies the barrier height Δ*E* = (126–251)*k_B_T* = (74–148) kcal/mol. In order to overcome such a barrier, one would have to hydrolyze 6–12 ATP molecules. Thus, in the absence of active processes, the double-membrane will remain in the sheet state even when *r_sheet_*/*r_rim_* becomes slightly larger than 5.1 and the energy barrier is somewhat reduced. However, when the sheet continues to grow up to the critical size *r* ^0^ *_sheet_*/*r_rim_* = 32/π≅10.2, see Equation 10 in Text S1 and Fig. 3, the energy barrier disappears and the flat state becomes unstable. For sizes equal to or larger than the critical size, the double-membrane sheet must undergo a transition towards the double-membrane organelle, see also Figs. S1 and S2.

In general, the bending and closure of the double-membrane sheet can proceed towards both sides of the sheet as indicated by the sequence of shapes above the energy landscapes in Fig. 3. In the absence of a preferred or spontaneous curvature, both closure pathways are degenerate since they are governed by the same energy landscape, which does not depend on the sign of the mean curvature *M*_1_, see Fig. 3. This “up-down” symmetry is still valid in the presence of nonvanishing preferred curvatures, *m*_1_, *m*_2_, and *m*_3_ as long as the two flat membrane segments of the sheet have the same preferred curvature, i.e., as long as *m*_1_ = *m*_2_. On the other hand, this symmetry is broken as soon as the two flat membrane segments have different preferred or spontaneous curvatures, i.e., for *m*_1_≠*m*_2_. The energy landscape then depends on the curvature asymmetry
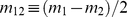
(5)as shown in Text S1 and Fig. S3.

### Dependence of critical sheet size on preferred curvatures

Bilayer membranes are usually asymmetric in the sense that their two leaflets differ in their molecular composition. As previously mentioned, this asymmetry leads to a preferred or spontaneous curvature of the membranes, which can be changed and regulated, for example, by desorption and adsorption of molecules from the surrounding solutions. Two examples are provided by the binding of proteins with BAR-domains or by the incorporation of lipids with large headgroups such as PI(3)P. For the double-membrane sheet, it is rather natural to distinguish the preferred curvature *m*_3_ of the sheet rim from the preferred curvatures *m*_1_ and *m*_2_ of the two faces of the sheet. These preferred curvatures have a strong influence on the critical sheet size. As shown in Equation 10 in Text S1, the critical sheet size depends only on two curvature parameters, on the preferred rim curvature *m*_3_ and on the curvature asymmetry *m*_12_.

**Figure 4:**
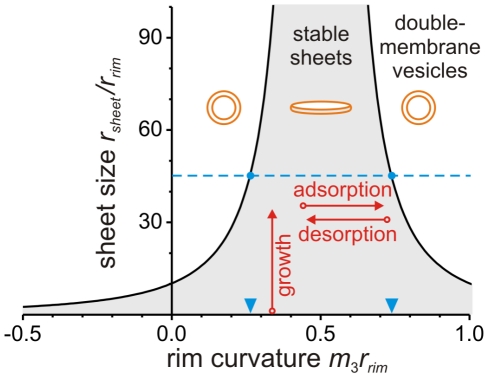
Stability diagram of double-membrane sheets as a function of preferred or spontaneous rim curvature *m*_3_ and sheet size *r_sheet_*. Both the rim curvature and sheet size are given in units of the rim curvature radius *r_rim_*. The sheets are (almost) symmetric in the sense that their two faces have similar preferred curvatures, *m*_1_≅*m*_2_ and the curvature asymmetry *m*_12_ is small compared to 1/*r_rim_*. The regime of stable sheets (gray area) is bounded by an instability line corresponding to the critical sheet size as described by Equation 10 in Text S1. The instability line has two branches for *m_3_r_rim_*<1/2 and *m_3_r_rim_*>1/2. For nonzero *m*_12_ the two branches meet at the maximal critical disk size as given by 2/*|m_12_|*. The latter size diverges for vanishing curvature asymmetry *m_12_* = 0, i.e., in this case, an arbitrarily large sheet remains stable. If the sheet reaches the instability line by lateral growth, protein adsorption and/or desorption at its rim (long arrows), it closes into a double-membrane vesicle. Sheets above the instability line are unstable and close into such vesicles as well. The broken horizontal line with *r_sheet_*/*r_rim_*≅45 corresponds to the autophagosome in Fig. 2C with diameter *r* ^0^ *_sheet_*≅900 nm and *r_rim_*≅20 nm. The intersection of the broken line with the two branches of the instability line determines the preferred or spontaneous rim curvature *m*_3_≅1/(76 nm) or 1/(28 nm) (arrowheads) of the unstable sheet that preceded the autophagosome in Fig. 2C.

The critical sheet size as given by Equation 10 in Text S1 defines the stability of double-membrane sheets as a function of sheet size *r_sheet_*, preferred rim curvature *m*_3_, and curvature asymmetry *m*_12_. The corresponding stability diagram is shown in Fig. 4 for (almost) symmetric sheets with small *m*_12_, where all length scales are measured in units of the rim curvature radius *r_rim_*. The stability diagram for asymmetric sheets with appreciable *m*_12_≠0 is shown in Fig. S4.

Inspection of Fig. 4 reveals that the regime of stable sheets is bounded by an instability line that depends on sheet size *r_sheet_*/*r_rim_* and preferred rim curvature *m*_3_ *r_rim_*. Both for small *m*_3_≪1/2*r_rim_* and for large *m*_3_≫1/2*r_rim_*, the critical sheet size is relatively small and the regime of stable sheets is rather narrow. For intermediate values *m*_3_≅1/2*r_rim_*, on the other hand, the critical sheet size is large and defines a rather wide regime of stable sheets. As we will discuss further below, the latter regime is relevant for the stability of flat organelles such as the cisternae in the Golgi apparatus and the extended sheets in the ER.

For DMOs with known spatial dimensions as determined experimentally, the stability diagram can be used to make predictions about the preferred or spontaneous curvatures of the membrane segments and thus yield information about processes such as protein adsorption to the different membrane surfaces. Such data can be obtained, e.g., from high resolution electron microscopy images [17]. In order to illustrate this point, let us consider the autophagosome shown in Fig. 2C which has a diameter of about 900 nm and the distance between the two bilayers is 2*r_rim_*≅40 nm. Let us assume that the preceding closure of the sheet did not involve significant lateral growth of this sheet. The diameter of the double-membrane organelle is then comparable to the critical sheet size *r* ^0^ *_sheet_*. This implies the reduced critical size *r* ^0^ *_sheet_*/*r_rim_*≅45. If we further assume that the sheet was (almost) symmetric and characterized by small curvature asymmetry *m*_12_, the intersection of the line *r_sheet_*/*r_rim_* = 45 with the two branches of the instability line in Fig. 4 leads to two possible values for the preferred rim curvature *m*_3_ as indicated by the two arrowheads in this figure. These two curvature values, 1/(76 nm) and 1/(28 nm), correspond to low and high critical concentrations of curvature generating molecules at the sheet rim, respectively. Conditions, for which *m*_3_<1/(76 nm) or *m*_3_>1/(28 nm), would induce sheet bending.

So far, we focused on the behavior of (almost) symmetric sheets with *m*_12_ close to zero. In general, the critical sheet size exhibits a maximum at the preferred rim curvature *m*_3_ = 1/2*r_rim_* for all values of the asymmetry *m*_12_. For small asymmetry, this maximum is rather sharp, see Fig. 4 and Fig. S4. This implies that the critical sheet size is very sensitive to small changes of the preferred rim curvature and/or the symmetry between the two faces of the sheet. Such small changes could be induced, e.g., by the adsorption and desorption of relatively small numbers of proteins. Large curvature asymmetries destabilize flat sheets significantly and even relatively small sheets will bend and close into double-membrane vesicles, see Fig. S4.

In summary, the main mechanism driving the closure of the double-membrane sheet is the competition between the bending energy and the effective rim tension (proportional to κ/*r_rim_*) arising from the strongly curved rim of the sheet. The growing sheet becomes unstable at a critical size, at which the energy barrier vanishes and the transition to a double-membrane organelle occurs spontaneously. We showed that a nonuniform composition of the membrane can easily modulate the size of the closed organelles and the direction of bending by influencing either the preferred curvature of the bilayer or the difference between the preferred curvatures of the two bilayers on both sides of the organelle. As discussed below, cells can regulate these asymmetries dynamically by proteins.

### Biological relevance

We propose that the mechanism described above is employed by cells to create double-membrane organelles. As the double-membrane sheet grows and/or the membrane composition changes by protein adsorption and desorption altering the preferred or spontaneous curvatures, the sheet can approach its critical size. The energy barrier for bending and closure into a double-membrane organelle is further and further reduced. The growing double-membrane sheet enters a metastable state, where even modest changes in the local environment are sufficient to overcome the energy barrier and induce a shape transition. When the energy barrier is of the order of *k_B_T*, thermal fluctuations or weak membrane-protein interactions are sufficient to initiate the shape transformation. Thus, important features such as the direction of membrane bending or the final size of the organelle can be adjusted by the cell with only minimal effort. In the present section, we compare predictions of our model with the available experimental data.

The double-membrane sheets observed during the sporulation of a yeast cell originate from small precursor vesicles [2]. The mechanism of membrane delivery to the phagophore is not known precisely, but fusion of small vesicles is one of the possible pathways [8], [18], [19], [20]. Vesicle fusion modifies the relative size *r_sheet_*/*r_rim_* of the double-membrane sheet. Both membrane area and volume are added, but the area always increases faster than the volume (consider the fusion of two spherical vesicles: the fused vesicle will not have a spherical shape, because the total area is larger than the area of a sphere with the new volume). Overall, the sheet size *r_sheet_* will increase during growth, while its thickness *r_rim_* may stay essentially constant or may even decrease. Thus, the relative size *r_sheet_*/*r_rim_* increases and the growing sheet will eventually attain its critical sheet size and close into a double-membrane vesicle.

The membrane supply to the organelle determines the growth rate of the double-membrane sheet. The rate of growth could be influenced by membrane fusion and probably other energy-dependent processes. Slow growth may kinetically trap the sheet close to the critical size flattening the energy landscape, see Fig. S2. At the critical size, this landscape exhibits a relatively flat plateau around the sheet state slowing down the sheet closure. Fast growing sheets will pass the critical size and can become large compared to this size.

It is difficult to estimate the time scales for sheet growth and closure from the available experimental data on the autophagosomal process, since this process has been primarily studied by electron microscopy. The combined process seems to be completed within about 15 minutes after induction [21]. Qualitative observations using confocal microscopy suggest that this time scale is dominated by the growth of the double-membrane organelle, whereas its closure is rather fast [22]. The latter conclusion is confirmed by considering the hydrodynamic dissipation of the elastic energy stored in the sheet.

For the parameter values used in Fig. 3, this stored elastic energy is 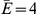 or *E* = 16πκ at the instability point, at which the double-membrane sheet becomes unstable. During the closure of the sheet, this energy will be dissipated within a volume of the order of *r_sheet_* ^3^. The corresponding time scale is proportional to η*r_sheet_* ^3^/κ where η is the dynamical viscosity of the surrounding medium. The proportionality factor includes a factor 1/2 since we consider here the bending of the double membrane, i.e., of two strongly coupled membranes, with effective bending rigidity 2κ. Using the dynamical viscosity of pure water, this time scale is found to be about 10^−2^ s for a sheet of radius 1 µm. In vivo, this time scale will be increased by the increased viscosity of the cytosol.

In the previous estimate, we have assumed that the elastic energy is primarily dissipated by hydrodynamic flow within the aqueous medium surrounding the double membrane. In general, one may envisage additional dissipative processes. One such process is provided by the flow within the thin water layer bounded by the double membrane, a flow that may be hindered by membrane undulations as proposed in Ref. [23], see also [24]. Another process that will contribute to the dissipation in our system is the hydrodynamic flow across the strongly curved rim of the double-membrane sheet. Indeed, during the closure of the double-membrane sheet into the double-membrane vesicle, membrane area must continuously flow from the inner to the outer membrane segment. The total area of this redistributed membrane is comparable to the initial area of the strongly curved membrane rim. The relative contributions of these different dissipative processes could be determined by explicit studies of the hydrodynamic flow during the closure of the double-membrane organelle.

Starvation or rapamycin treatment stimulate the extensive formation of autophagosomes and increase their growth rate significantly as compared to that of basal autophagy [21]. Recent experiments on neuronal autophagosomes [25] show that induced autophagosomes are generally larger in size compared to basal autophagosomes. These results suggest that an increase of the autophagosomal growth rate increases the size of the final autophagosome. During sporulation of *Schizosaccharomyces pombe*, the forespore membrane in the wild type grows fast to large sizes and closes fast [12]. The spo3 mutant in contrast, shows impaired membrane growth. The final forespore membranes of the mutant are considerably smaller and it takes them significantly longer to close. These two distinct cellular processes, both based on transient double membrane organelles, support dynamical aspects of the mechanism proposed here, namely that fast growing double membranes lead to large organelles, which close fast. This suggests that by simply increasing the autophagosomal growth rate under acute stress conditions the autophagosomal load can be brought faster to the lysosomal decomposition and larger volumes can be degraded, making the autophagy process much more efficient.

Our theory as described above implies that an uneven distribution of membrane components or an asymmetric insertion of molecules strongly affect the morphological transformation of the sheet. Examples for such curvature modifying processes include adsorption of proteins with BAR-domains [26], palmitoylation of peripheral proteins [27], ubiquitin-like conjugations, and phosphorylation of membrane components. For example, autophagosome formation requires class III PI3-kinase activity and the two ubiquitin-like conjugation systems Atg8 and Atg12. The latter systems relocate cytosolic Atg8 to the membrane by covalently binding it to the membrane lipid phosphatidylethanolamine [28], [29], thus changing the preferred curvature of the membrane. The Atg16L complex, composed of Atg5–12 and Atg16, is preferentially associated with the external membrane of the autophagosome [13], [28] suggesting that this complex imposes an asymmetry in the preferred curvature of the inner and outer (or upper and lower) membranes, i.e., *m*_1_≠*m*_2_. According to our theory, this membrane asymmetry is sufficient to establish the bending direction of the phagophore already at an early stage. Since strong asymmetries can reduce the critical size and modulate the bending direction, the decrease of the autophagosome size at high concentrations of Atg16L or related proteins is plausible, compare Fig. S3.

Molecules may absorb at the rim of the sheet locally. Lipids such as PI(3)P localize at the rim of the growing sheet [30]. Proteins involved in fission of double-membrane organelles, as proposed for the lipidated Atg8 (Atg8-PE), must also reside at the rim in order to fulfill their function [31]. Atg8-PE is known to regulate the size of autophagosomes, but the regulatory mechanism of the protein is not understood [32]. If large numbers of molecules adsorb at the rim, the preferred or spontaneous curvature *m*_3_ increases locally. We showed that such an increase leads to a larger critical size and larger final autophagosome size provided the rim curvature *m*_3_<1/2*r_rim_*. In contrast, a decrease in *m*_3_ resulting from a reduced concentration of the adsorbing molecules would result in a smaller organelle size. Thus our model suggests that the general decrease of autophagosome size observed during knock down of Atg8 [32] is caused by a concentration dependent decrease of the preferred rim curvature *m*_3_. In a similar way, the deconjugation of Atg8 from the membrane during autophagosome formation [32] can bring the double-membrane sheets across the stability line and induce sheet bending, see Fig. 4.

Several types of specific autophagy are known, some of them being named after the type of the enwrapped substrate such as peroxisomes (pexophagy) or mitochandria (mitophagy). The degradation pathways involve a specific interaction of the phagophore membrane with the substrate. One example is the sequestration of inclusion bodies containing the protein p62. The latter protein interacts directly with Atg8 and its homologues bound to the phagophore membrane [33], [34], [35]. The adhesion of the substrate to one side of the sheet can reduce the energy required for sheet closing, see Text S2 and Fig. S5 for details. Therefore, the final autophagosome size can be smaller in selective autophagy compared to unspecific autophagy.

The shape of some organelles such as cisternae in the Golgi apparatus and parts of the ER represent flat double-membrane sheets with reduced sizes in the range 15<*r_sheet_*/*r_rim_*<40 [36], [37]. In contrast to transient DMOs, these organelles do not bend to form double-membrane vesicles but are stable as flat sheets. The results displayed in Fig. 4 and Fig. S4 suggest that sheets with such dimensions are stable only for a certain range of preferred or spontaneous rim curvatures *m*_3_ and for a relatively small curvature asymmetry *m*_12_. Recently, reticulons and DP1/Yop1p proteins were found to stabilize ER-sheets by adsorbing at the rim [37], [38], thus changing the rim preferred curvature *m_3_* and stabilizing the flat sheet morphology as proposed by our model. Consequently, in the absence of these proteins highly curved membrane domains of the ER tend to vanish [39]. The influence of curvature asymmetries in the ER sheets on their stability has not been studied so far. The Golgi cisternae are morphologically similar to the ER-sheets. This implies that analogous mechanisms may regulate the preferred membrane curvatures in the two organelles. These mechanisms, along with the structural support provided by the cytoskeleton [40], may play a role in the stability of the Golgi cisternae and the ER sheets.

### Conclusions

The mechanisms for bending of double-membrane sheets into double-membrane organelles have not been identified until now. This applies not only to yeast sporulation [2] and viral infections [3], but also to the autophagic pathways [4], [8], [41]. Active mechanisms for generating bilayer curvature in cells such as molecular motors, cytoskeletal polymerization or lipid flippases [42], [43] do not seem to apply. Scaffold or coat proteins have also been hypothesized to play a role in, for example, phagophore bending [13], [44]. However, COPII coat proteins have been found to cover typically only small vesicles with sizes in the range 60–100 nm [45], while DMO sizes can vary in the broader range from 70 nm for double membrane vesicles [6] to 7 µm for autophagosomes [46]. Furthermore, no protein coats on autophagosomes have been observed in electron microscopy studies so far. Instead, in freeze-fracture images autophagosomal membranes appear very smooth, see Fig. 2D. Remarkably, approximately 30% of autophagosomes lack any integral proteins [47] and all other autophagosomal membranes have a density of integral proteins several orders of magnitude lower than that in other organelles. The amount of peripheral proteins at the autophagosomal membrane is low as well [47]. Similarly, viruses employ only a very limited number of proteins to form double-membrane vesicles, the number being limited by the small size of the viral capsids. These observations indicate that only a small amount of membrane proteins may be required for the regulation of curvature in double-membrane organelles.

Here, we propose a novel mechanism for curvature generation, according to which sufficiently large double-membrane sheets transform into double-membrane organelles. This mechanism can be understood from the interplay between the local preferred curvature of the membrane, arising, e.g., from a different protein/lipid composition in the two leaflets of the bilayer, and the membrane's bending rigidity, which leads to an effective rim tension along the highly curved segments of the double membrane. The closure of this membrane can therefore be induced by protein adsorption or recruitment, which changes the preferred membrane curvature, or by lateral growth of the double- membrane sheet, which increases the energy of the strongly curved rim. When the dimensions of the sheet are close to the critical size, the sheet becomes unstable and transforms into a double-membrane organelle, see the stability diagram in Fig. 4.

Autophagy and sporulation are induced by extreme environmental conditions such as starvation and stress. Both processes play a role in cell survival [1], [7]. In such a critical situation, the cell should try to avoid pathways that are energetically expensive. The curvature generation mechanism described here reduces the cellular expenses needed to form a transient double-membrane organelle: the main driving force is provided by the organelle size and the regulation requires minimal machinery. Thus, the proposed mechanism is evolutionary advantageous.

The formation of cellular double-membrane sheets is complex and regulated by a network of molecular processes [48]. Important aspects of these processes are not fully characterized, especially concerning the formation of double-membrane vesicles during viral infections [3]. The membrane sheets can have continuous membrane connections with other organelles via one or even multiple contacts [22], [49], [50], and a shape transformation can be sterically hindered, for example, by large substrates or unrelated organelles. All of these factors will influence the dynamics of morphological transitions in the cell and may also lead to distorted organelle shapes (as observed for omegasomes) compared to the axisymmetric, cup-like intermediate shapes considered in our theory. Nevertheless, we showed that the rim energy plays the critical role for the closure of double-membrane sheets into double-membrane organelles.

The theory for the bending of double membranes presented here provides a new quantitative framework for the interpretation of shape transformations in cellular organelles. We discuss several mechanisms for the regulation of these processes, and demonstrate that our results are in agreement with available experimental data. Finally, we emphasize that the mechanism of double-membrane sheet bending described here is quite different from well-established cellular mechanisms for modulating the morphology of single-membrane sheets or for forming intracellular vesicles, which involve active processes.

## Supporting Information

Text S1 **Continuous deformation of a flat double-membrane sheet into a closed vesicle via cup-shaped intermediates.**
(DOC)

Text S2 **Wrapping a double membrane around an adhesive ‘particle’**
(DOC)

Figure S1 **Geometrical parameters of a cup-shaped intermediate.** The radius of the cup *r_cup_* and the curvature radius *R* are shown. The lower (dashed blue), upper (solid green) and the rim (solid red) segments are indicated with 1, 2 and 3, respectively.
(TIF)

Figure S2
**Reduced energy** 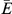 **of double-membrane organelles as a function of the reduced curvature** ***r_sheet_M*** **_1_ for** ***m*** **_12_ = 0 and** ***m*** **_3_ = 0.** At and above the critical size, *r_sheet_*/*r_rim_* = 10.2, no barrier exists anymore, the closed organelle is the shape of minimal energy and bending of the flat sheet is energetically favorable. The points where the energy is decreased by 1% of the energy of the initial sheet are marked with a cross (x). Small organelles close to the critical size can deform strongly without considerable change in the bending energy, while large organelles reduce their bending energy already at comparably small deformations. Thus, a large sheet has a high probability to close within a short time. Small sheets, even if larger than their critical size, will close after a considerable lag-time.(TIF)

Figure S3
**Reduced bending energy of double-membrane shapes,** 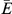 **, as a function of the reduced curvature** ***r_sheet_M*** **_1_ calculated for different values of the curvature asymmetry** ***m*** **_12_.** An asymmetrical distribution of molecules on both sides of the double membrane changes the curvature asymmetry *m*_12_ and favors a certain direction of bending. The reduced energy is plotted for different values of *m*_12_, dimensionless sheet size *r_sheet_*/*r_rim_* = 7.65 and preferred or spontaneous rim curvature *m*_3_ = 0. For *m*_12_ = 0 (solid curve) as presented in Fig. 4 in the main text, the probabilities for upward or downward curving are equal. Nonzero values of *m*_12_ break this “up-down” symmetry of the energy profile.(TIF)

Figure S4 **Dependence of the critical size** ***r*** **^0^** ***_sheet_*** **of the sheet as a function of the preferred or spontaneous rim curvature** ***m*** **_3_ for different values of the curvature asymmetry** ***m*** **_12_.** All quantities are given in units of the rim curvature radius *r_rim_*. The diagram displays four instability lines corresponding to four different values of the curvature asymmetry *m*_12_ *r_rim_*. The regions below and above one of the instability lines correspond to conditions for stable double-membrane sheets and vesicles, respectively. For *r* ^0^ *_sheet_*/*r_rim_*≅45 (black solid line) corresponding to the autophagosome size with *r_rim_* = 20 nm in Fig. 2C, the rim curvature *m*_3_≅1/(76 nm) or 1/(28 nm) for the case of symmetric sheets with *m*_12_ = 0, see black arrowheads, while for a sheet with curvature asymmetry *m*_12_ *r_rim_* = 0.02, the rim curvature *m*_3_≅1/(54 nm) or 1/(32 nm), see green arrowheads.
(TIF)

Figure S5 **Wrapping a double membrane around an adhesive ‘particle’.** The adhesion between the surface of the ‘particle’ (grey) and the upper membrane of the double-membrane sheet (orange) is mediated by receptors attached to the ‘particle’ surface and ligands anchored at the double membrane. When these two surfaces are sufficiently close, the ligands and the receptors form molecular bonds as indicated by the red-green adhesion (or contact) areas. (A) In its flat state, the double-membrane sheet has a relatively small adhesion area with the ‘particle’. (B, C) As the sheet starts to bend towards the ‘particle’, the adhesion area increases. (D) The adhesion area is now equal to the surface area of the ‘particle’, which is fully enwrapped by the double membrane. This membrane now forms a spherical vesicle with a small neck.
(TIF)
