# Supplementary material for: Curvature of Double-Membrane Organelles Generated by Changes in Membrane Size and Composition
Source: PLoS One. 2012 Mar 12;7(3):e32753. doi: 10.1371/journal.pone.0032753 (PMC3299685; doi:10.1371/journal.pone.0032753)
Supplement: Text S1 — Continuous deformation of a flat double-membrane sheet into a closed vesicle via cup-shaped intermediates. (DOC) [file pone.0032753.s001.doc]

# Curvature of double-membrane organelles generated by changes in membrane size and composition

Roland L. Knorr, Rumiana Dimova*, Reinhard Lipowsky

Max Planck Institute of Colloids and Interfaces, Science Park Golm, 14424 Potsdam, Germany

*Corresponding author: dimova@mpikg.mpg.de

Tel: +49 331 567 9615; Fax: +49 331 567 9612

## Text S1

### Continuous deformation of a flat double-membrane sheet into a closed vesicle via cup-shaped intermediates

In the limit of large *r_sheet_* >> *r_rim_*, the bending energy of a sheet with non-zero preferred (or spontaneous) curvatures *m*_1_, *m*_2_, and *m*_3_ of the three membrane zones 1, 2, and 3, see Supporting Fig. S1, has the form

$E_{sheet}\approx\pi^{2}\kappa\frac{r_{sheet}}{r_{rim}}\left(1-2m_{3}r_{rim}\right)^{2}+2\pi\kappa r_{sheet}^{2}\left(m_{1}^{2}+m_{2}^{2}\right)$ (1)

If only the rim of the sheet has a non-zero preferred curvature *m*_3_ ≠ 0 and the preferred curvatures *m*_1_ and *m*_2_ of two faces of the sheet vanish, the second term of expression (1) vanishes.

In the limit of large *R* >> *r_rim_*, the bending energy of a vesicle or an organelle with distinct preferred curvatures of the inner and outer membranes, i.e., with *m*_1_ ≠ *m*_2_ is given by

$E_{ves}\approx8\pi\kappa\left[1+\left(1-2Rm_{12}\right)^{2}+2R^{2}m_{1}m_{2}\right]$ (2)

where we used the curvature asymmetry *m*_12_ = (*m*_1_ – *m*_2_)/2; see also Supporting Fig. S1. The curvature asymmetry vanishes if the double-membrane sheet exhibits an “up-down” symmetry between its two faces, i.e., the outer leaflets of the two apposing membranes have the same structure and composition and likewise for the two inner leaflets.

The total bending energy of a cup-shaped organelle can be decomposed according to

$E=E_{1}+E_{2}+E_{3}$, (3)

where *E*_1_ and *E*_2_ arise from the bending of the two, initially flat surfaces (corresponding to *E_ves_* for the closed organelle) and *E*_3_ is the contribution arising form the strongly bent rim (corresponding to *E_sheet_* for the flat sheet conformation). In the limit of large *R* >> *r_rim_*, the sum *E*_1_ + *E*_2_ for the cup-shaped organelle with the total surface area *A* ≈ 2π*r_sheet_*^2^ is given by [1]

$E_{1}+E_{2}\approx4\pi\kappa r_{sheet}^{2}\left[\left(M_{1}-m_{12}\right)^{2}+m_{12}^{2}+m_{1}m_{2}\right]$. (4)

Here, we consider a cup-shaped organelle without an “up-down” symmetry between its two faces, which implies non-zero curvature asymmetry *m*_12_ ≠ 0.

The rim energy *E*_3_ can be expressed in terms of (i) the cup radius *r_cup_* as introduced in Supporting Fig. S1 and (ii) the effective rim tension

$\lambda_{eff}\equiv\frac{E_{sheet}}{2\pi r_{sheet}}=\frac{\pi\kappa}{2r_{rim}}\left(1-2m_{3}r_{rim}\right)^{2}$. (5)

Then for *r_cup_* >> *r_rim_*, a cup-shaped intermediate with rim length 2π*r_cup_* will be characterized by the rim energy

$E_{3}\approx2\pi\lambda_{eff}r_{cup}$ (6)

Taking into account that $r_{cup}=r_{sheet}\sqrt{1-\left(r_{sheet}M_{1}/2\right)^{2}}$, we obtain

$E_{3}\approx\pi^{2}\kappa\frac{r_{sheet}}{r_{rim}}\left(1-2m_{3}r_{rim}\right)^{2}\sqrt{1-\left(r_{sheet}M_{1}/2\right)^{2}}$. (7)

For convenience we introduce the dimensionless bending energy

$\overline{E}=E/4\pi\kappa-r_{sheet}^{2}\left(m_{1}+m_{2}\right)^{2}/4$. (8)

The second term in this expression is independent of the mean curvature *M*_1_ of the cup-shaped vesicle. A combination of the expressions (3), (4) and (7) leads to

$\overline{E}\approx r_{sheet}^{2}\left(M_{1}-m_{12}\right)^{2}+\pi\frac{r_{sheet}}{4r_{rim}}\left(1-2m_{3}r_{rim}\right)^{2}\sqrt{1-\left(r_{sheet}M_{1}/2\right)^{2}}$, (9)

which depends on four dimensionless quantities: the rescaled mean curvature *r_sheet_* *M*_1_, the rescaled curvature asymmetry *r_sheet_ m*_12_ , and the rescaled preferred (or spontaneous) rim curvature *m*_3_ *r_rim_* as well as the ‘aspect ratio’ *r_sheet_*/*r_rim_*.

As explained in the main text, the elastic energy density of the membrane also includes a term proportional to the Gaussian curvature, which is not taken into account here, since this term gives a constant energy contribution during the whole closure process of the double membrane up to the fission of the narrow membrane neck. Thus, the bending energy (9) represents the energy landscape for the transformation of the double-membrane sheet into a double-membrane vesicle with a tiny membrane neck. The latter shape represents a limit shape: it consists of two concentric, spherical membranes which are connected by a narrow neck, the area of which is negligible compared to the area of the spherical membranes.

In order to analyze the bending energy as given by (9), it is convenient to choose fixed values for the three parameters *r_sheet_ m*_12_, *m*_3_ *r_rim_* and *r_sheet_*/*r_rim_* and to plot the energy (9) as a function of the mean curvature *r_sheet_* *M*_1_. In this manner, we calculated the energy landscapes as displayed in Fig. 3 and Supporting Fig. S2 for *m*_12_ = 0 as well as in Supporting Fig. S3 for *m*_12_ > 0.

The energy landscape for *m*_12_ = 0 as shown in Supporting Fig. S2 illustrates the slow kinetics of the double-membrane sheet when its size is close to the critical size. In the latter case, the energy landscape exhibits a large, flat plateau around the sheet state, and even pronounced shape changes corresponding to the curvature range ˗1 < *r_sheet_M*_1_ < 1 do not lead to a significant reduction of the bending energy (the crosses in Supporting Fig. S2 correspond to a decrease of the bending energy by 1%). These energy plateaus indicate that the sheet will bend and close relatively slowly. On the other hand, fast growing sheets will pass the critical size and can become large compared to this size. In this state, relatively small values of the mean curvature *r_sheet_M*_1_ will lead to a significant decrease in the bending energy of the double membrane and to a fast closure of the sheet.

Some examples for energy landscapes with non-zero curvature asymmetry *m*_12_ > 0 are depicted in Supporting Fig. S3. For *m*_12_ > 0, the “up-down” symmetry of the energy landscape is broken in contrast to the symmetric landscapes in Fig. 3 and in Supporting Fig. S2.

Having obtained an expression for the energy of the double-membrane shapes, we can now proceed to determine the reduced critical sheet size *r*^0^*_sheet_/r_rim_*. Using the same line of reasoning as in [1], we then obtain the expression

$\frac{r_{sheet}^{0}}{r_{rim}}=\frac{32}{\pi}\left(\left|1-2m_{3}r_{rim}\right|^{4/3}+\left|16m_{12}r_{rim}/\pi\right|^{2/3}\right)^{-3/2}$ (10)

for the reduced critical sheet size which shows that this size depends only on two parameters, the preferred (or spontaneous) rim curvature *m*_3_*r_rim_* and the curvature asymmetry *m*_12_*r_rim_*. For a given value of *m*_12_*r_rim_*, the critical sheet size as given by (10) has two branches for *m*_3_*r_rim_* < 1/2 and *m*_3_*r_rim_* > 1/2. These two branches meet at *m*_3_*r_rim_* = 1/2 with a cusp-like singularity, reflecting the exponent 4/3 in (10). For *m*_3_*r_rim_* = 1/2, the critical sheet size attains its maximal value 2/|*m*_12_|, which diverges for small values of *m*_12_. The expression (10) for the critical size of the double-membrane sheet is plotted in Fig. 4 for the symmetric case with *m*_12_ = 0 and in Supporting Fig. S3 for *m*_12_ ≠ 0. For double-membrane spherical organelles with the reduced size *r*^0^*_sheet_*/*r_rim_* ≅ 45 as considered in the main text, the reduced rim curvatures for symmetric sheets with *m*_12_ = 0 and for sheets with curvature asymmetry *m*_12_*r_rim_* = 0.02 are indicated in the figure caption of Supporting Fig. S4. Inspection of this figure also reveals that sheets with size *r*^0^*_sheet_*/*r_rim_* ≅ 45 and large curvature asymmetry *m*_12_*r_rim_* > 2/45 will never be stable irrespective of the value of the preferred (or spontaneous) curvature *m*_3_ *r_rim_* of the sheet rim.

## References

1. Lipowsky R (1992) Budding of membranes induced by intramembrane domains. Journal de Physique II 2: 1825-1840.
