# Supplementary material for: Curvature of Double-Membrane Organelles Generated by Changes in Membrane Size and Composition
Source: PLoS One. 2012 Mar 12;7(3):e32753. doi: 10.1371/journal.pone.0032753 (PMC3299685; doi:10.1371/journal.pone.0032753)
Supplement: Text S2 — Wrapping a double membrane around an adhesive ‘particle’ (DOC) [file pone.0032753.s002.doc]

# Curvature of double-membrane organelles generated by changes in membrane size and composition

Roland L. Knorr, Rumiana Dimova*, Reinhard Lipowsky

Max Planck Institute of Colloids and Interfaces, Science Park Golm, 14424 Potsdam, Germany

*Corresponding author: dimova@mpikg.mpg.de

Tel: +49 331 567 9615; Fax: +49 331 567 9612

## Text S2

### Wrapping a double membrane around an adhesive ‘particle’

Specific autophagy of an intracellular ‘particle’ is mediated by attractive interactions between ligand molecules attached to the particle surface and receptor molecules anchored within the double membrane. Since this interaction is attractive, it reduces the total energy of the combined system which now has the form

$E=E_{1}+E_{2}+E_{3}+E_{ad}$ (11)

with the adhesion energy *E_ad_* = *WA_ad_*. The adhesion energy per unit area, *W*, is taken to be negative and *A_ad_* is the adhesion (or contact) area. A receptor can bind its ligand provided the two molecules are sufficiently close to each other. If a flat membrane sheet approaches the ‘particle’, the adhesion area *A_ad_* for initial binding is relatively small and thus will not contribute significantly to the total energy of the sheet *E*, see Supporting Fig. S5, which illustrates a case of weak adhesion. As the sheet bends towards the ‘particle’, the adhesion area increases and the adhesion energy *E_ad_* makes a larger negative contribution to the total energy (11) of the double membrane/‘particle’ system, see Supporting Fig. S5. Therefore, the adhesion energy acts to reduce the energy barrier for the closure of the double-membrane sheet and, thus, to facilitate this closure process.
