# Supplementary figures and images for: Curvature of Double-Membrane Organelles Generated by Changes in Membrane Size and Composition
Source: PLoS One. 2012 Mar 12;7(3):e32753. doi: 10.1371/journal.pone.0032753 (PMC3299685; doi:10.1371/journal.pone.0032753)

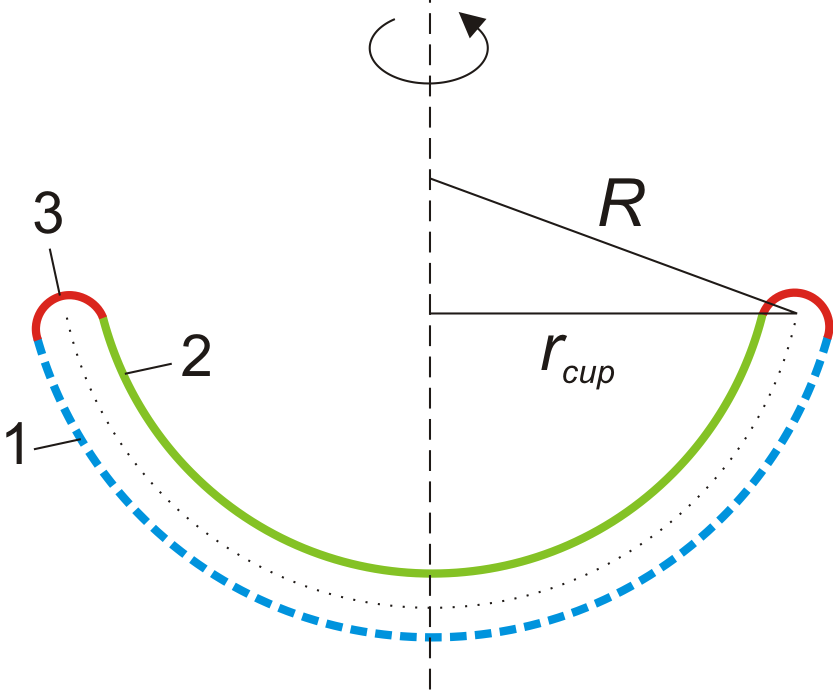

Supplement: Figure S1 — Geometrical parameters of a cup-shaped intermediate. The radius of the cup rcup and the curvature radius R are shown. The lower (dashed blue), upper (solid green) and the rim (solid red) segments are indicated with 1, 2 and 3, respectively. (TIF) [file pone.0032753.s003.tif]

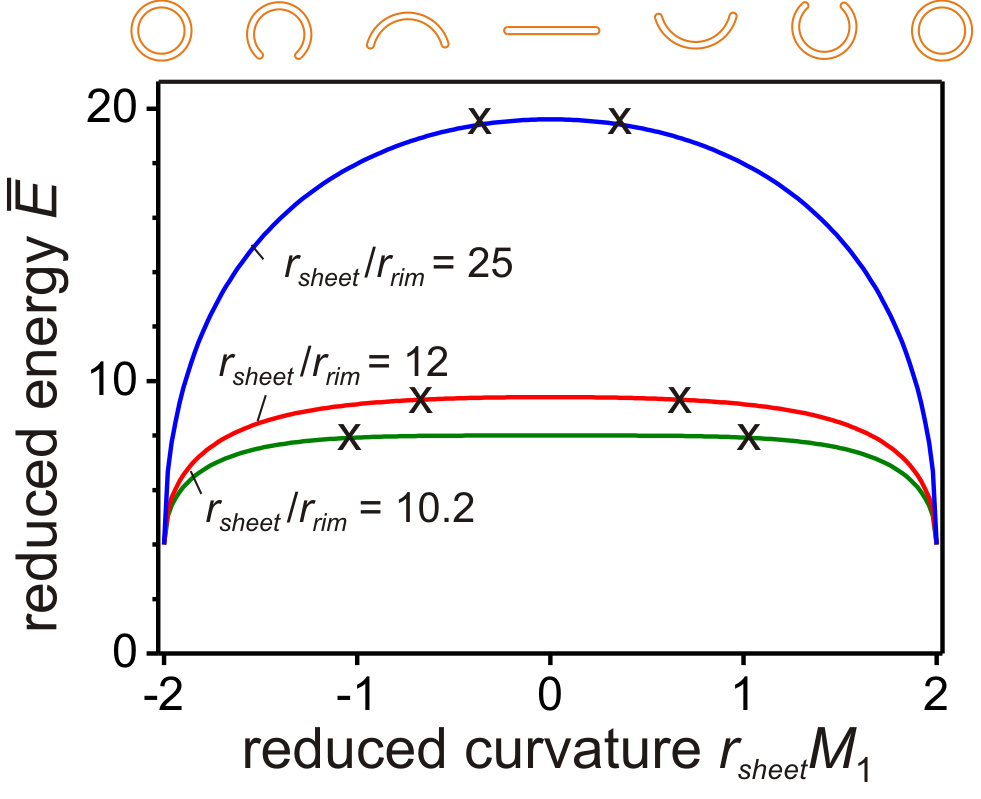

Supplement: Figure S2 — Reduced energy of double-membrane organelles as a function of the reduced curvature rsheetM 1 for m 12 = 0 and m 3 = 0. At and above the critical size, rsheet/rrim = 10.2, no barrier exists anymore, the closed organelle is the shape of minimal energy and bending of the flat sheet is energetically favorable. The points where the energy is decreased by 1% of the energy of the initial sheet are marked with a cross (x). Small organelles close to the critical size can deform strongly without considerable change in the bending energy, while large organelles reduce their bending energy already at comparably small deformations. Thus, a large sheet has a high probability to close within a short time. Small sheets, even if larger than their critical size, will close after a considerable lag-time. (TIF) [file pone.0032753.s004.tif]

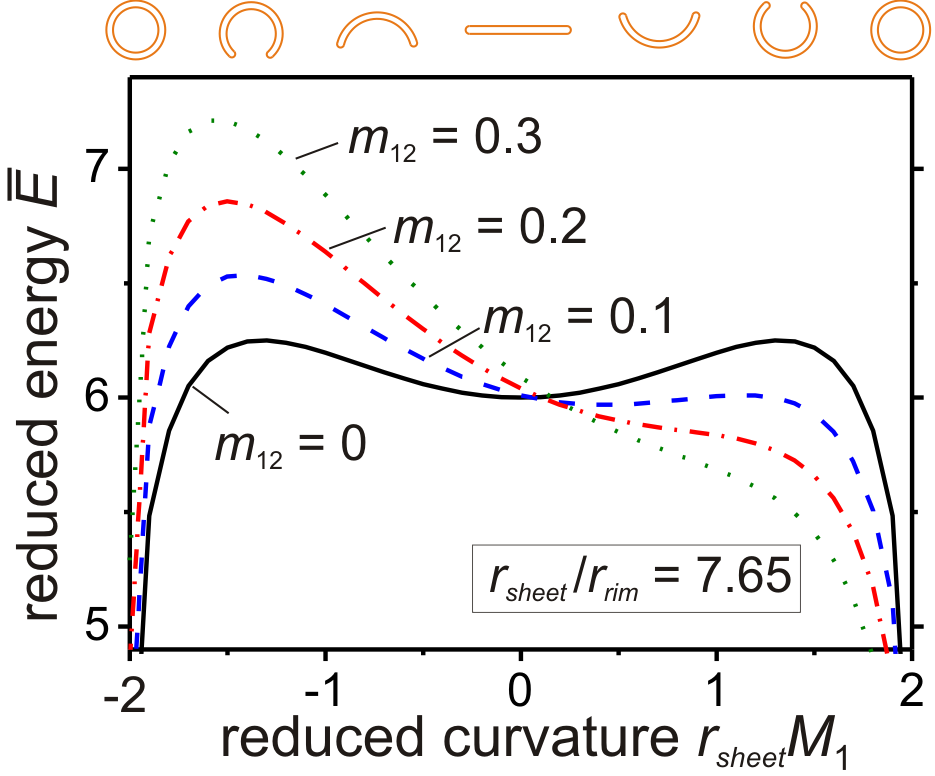

Supplement: Figure S3 — Reduced bending energy of double-membrane shapes, , as a function of the reduced curvature rsheetM 1 calculated for different values of the curvature asymmetry m 12. An asymmetrical distribution of molecules on both sides of the double membrane changes the curvature asymmetry m 12 and favors a certain direction of bending. The reduced energy is plotted for different values of m 12, dimensionless sheet size rsheet/rrim = 7.65 and preferred or spontaneous rim curvature m 3 = 0. For m 12 = 0 (solid curve) as presented in Fig. 4 in the main text, the probabilities for upward or downward curving are equal. Nonzero values of m 12 break this “up-down” symmetry of the energy profile. (TIF) [file pone.0032753.s005.tif]

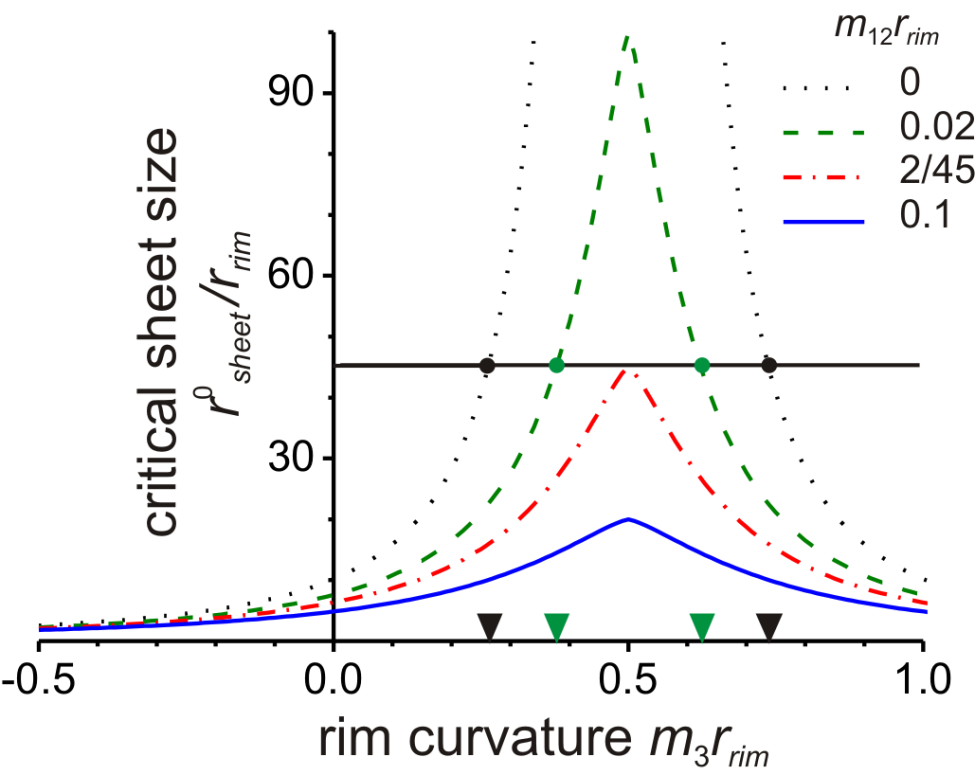

Supplement: Figure S4 — Dependence of the critical size r 0 sheet of the sheet as a function of the preferred or spontaneous rim curvature m 3 for different values of the curvature asymmetry m 12. All quantities are given in units of the rim curvature radius rrim. The diagram displays four instability lines corresponding to four different values of the curvature asymmetry m 12 rrim. The regions below and above one of the instability lines correspond to conditions for stable double-membrane sheets and vesicles, respectively. For r 0 sheet/rrim≅45 (black solid line) corresponding to the autophagosome size with rrim = 20 nm in Fig. 2C, the rim curvature m 3≅1/(76 nm) or 1/(28 nm) for the case of symmetric sheets with m 12 = 0, see black arrowheads, while for a sheet with curvature asymmetry m 12 rrim = 0.02, the rim curvature m 3≅1/(54 nm) or 1/(32 nm), see green arrowheads. (TIF) [file pone.0032753.s006.tif]

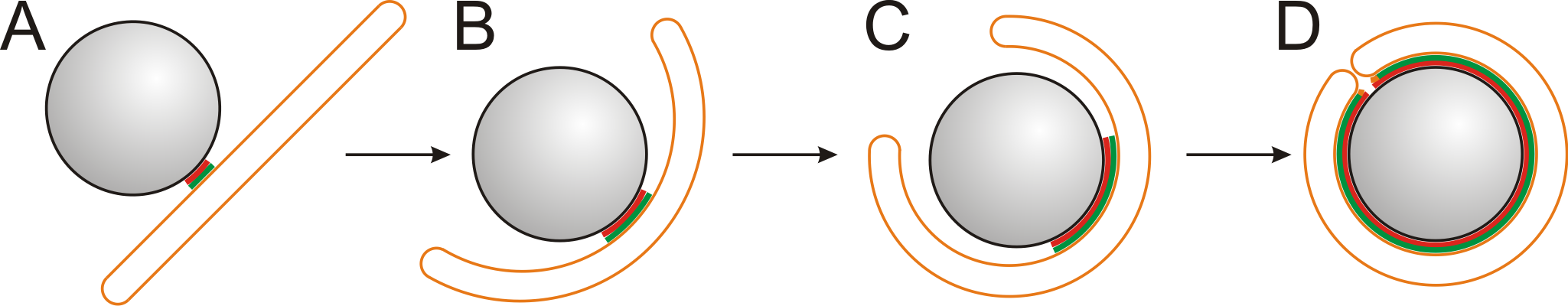

Supplement: Figure S5 — Wrapping a double membrane around an adhesive ‘particle’. The adhesion between the surface of the ‘particle’ (grey) and the upper membrane of the double-membrane sheet (orange) is mediated by receptors attached to the ‘particle’ surface and ligands anchored at the double membrane. When these two surfaces are sufficiently close, the ligands and the receptors form molecular bonds as indicated by the red-green adhesion (or contact) areas. (A) In its flat state, the double-membrane sheet has a relatively small adhesion area with the ‘particle’. (B, C) As the sheet starts to bend towards the ‘particle’, the adhesion area increases. (D) The adhesion area is now equal to the surface area of the ‘particle’, which is fully enwrapped by the double membrane. This membrane now forms a spherical vesicle with a small neck. (TIF) [file pone.0032753.s007.tif]
